# Treatment of c-kit positive adenoid cystic carcinoma of the tongue: A case report

**DOI:** 10.3892/ol.2014.2075

**Published:** 2014-04-16

**Authors:** MASSIMO MESOLELLA, AMALIA LUCE, ANNA MARINO, MICHELE CARAGLIA, FILIPPO RICCIARDIELLO, MAURIZIO IENGO

**Affiliations:** 1 Ear, Nose and Throat Department, University ‘Federico II’, Naples I-80138, Italy; 2 Department of Biochemistry, Biophysics and General Pathology, Second University of Naples, Naples I-80138, Italy

**Keywords:** adenoid cystic carcinoma, c-kit, cylindroma, lymph nodes, salivary gland neoplasm, tongue

## Abstract

Adenoid cystic carcinoma (ACC) or ‘cylindroma’ is a malignant tumor that often occurs in the areas of the head and neck, affecting the secretory glands and the major and minor salivary glands. The present study describes a case of a patient who presented with a posterior tongue lesion. The case is of a 71-year-old female with an asymptomatic volume growth of the posterior left tongue perceived 8 months prior, and neoplastic cells positive for c-kit. A computed tomography of the head and neck showed asymmetry of the base of the tongue, which was enlarged in the left portion. A physical examination revealed a nodule on the posterior left tongue of ~3 cm in diameter, while the cervical lymph node chain had a normal size and consistency. Surgical exeresis of the tongue lesion and cervical lymph node dissection were performed. Subsequent to surgical removal of the cancer cells and adjuvant radiotherapy, the patient showed excellent health, although the follow-up remains in progress. ACC, one of the most biologically destructive tumors of the head and neck, is locally aggressive and gives rise to distant metastases. The tongue is the place of origin in 3.4–17.1% of cases. The treatment for ACC consists of primary surgical resection with adjuvant radiotherapy. To prevent the risk for distant metastasis, it is necessary to remove the first echelon nodes and monitor the patient with a long-term follow-up.

## Introduction

In 1859, Billroth (1) termed adenoid cystic carcinoma (ACC) as cylindroma, and then the lesion was designated as ACC by Foote and Frazell in 1953 (2). ACC is a malignant tumor that originates in the salivary glands and represents ~1–4% of all malignant neoplasms of the head and neck and ≤5–15% of all malignant salivary gland neoplasms (3–5). It constitutes ~29.6% of minor salivary gland tumors (3), and the most common minor salivary gland site is the hard palate, followed by the base of the tongue (4.4%) (6–9). ACC is characterized by slow growth, local recurrence, perineural invasion and potential to produce distant metastases, mainly to the lungs and bones (3,6). The peak incidence occurs predominantly among females between the 5th and 7th decades of life (3,10). The current study presents a rare case of a 71-year-old female patient with a c-kit positive posterior tongue ACC treated with surgery and adjuvant radiotherapy. The patient provided written informed consent.

## Case report

A 71-year-old female presented at the ‘Federico II’ University Hospital (Naples, Italy) with an asymptomatic volume growth on the posterior left tongue, perceived 8 months previously. The patient did not have a history of smoking or alcohol consumption, and had an insignificant past medical history, with the exception of type II diabetes mellitus treated with oral hypoglycemic agents. The clinical examination revealed a nodule on the posterior left tongue of ~3 cm in diameter with a similar color to the buccal mucosa, while the cervical lymph node chain had a normal size and consistency. A computed tomography of the head and neck performed with administration of contrast medium showed asymmetry of the base of the tongue, enlarged in the left portion, without visibility of areas of densitometric alteration, and a few small lateral cervical lymph nodes of ~1 cm in diameter bilaterally. A transoral exeresis of the tongue lesion emptying cervical lymph nodes at levels I–III ipsilateral to the tumor was performed. During the intervention, a tracheotomy was carried out to ensure breathing, which could be compromised by a postoperative edema of the tongue, and also left lingual artery ligation, to reduce bleeding. Histopathological analysis demonstrated a diffuse infiltration by ACC, cribriform subtype; the tumor was absent on the margins of excision and the neoplastic cells were focally positive to immunohistochemical staining for c-kit. All lymph nodes resulted as reactive. A nasogastric tube was removed 10 days after surgery.

The patient then underwent postoperative radiotherapy and is currently under follow-up, demonstrating good health 12 months after the intervention.

## Discussion

ACC is one of the most biologically harmful and incalculable tumors of the head and neck (5), is locally aggressive and is characterized by infiltrative growth and perineural spread, leading to high recurrence levels, despite aggressive surgical resection and late metastasis, with patient mortality between 10 and 20 years after the initial treatment (5,9,11,12). The most common site of distant metastases is the lung, although bone, liver, kidney and prostate have also been reported (12).

ACC of the floor of the mouth and tongue requires a closer follow-up compared with other histiotypes. ACC of the tongue is rare and an early diagnosis is important as these are slow-growing tumors with diffuse invasion. Among ACC of the head and neck region, the tongue is the site of origin in 3.4–17.1% of the cases. A study by Luna-Ortiz *et al* (11) demonstrated that among their 68 patients with a diagnosis of head and neck ACC, eight cases (seven female and one male) were found to exhibit ACC of the tongue (11.7% of all head and neck ACCs), six at the base and two in the mobile tongue (Table I). When located in the tongue, the clinical course is usually asymptomatic, with gradual submucosal tumoral growth, with a diagnostic delay estimated from 6 months to 8 years (11,13). In the presented case of the current study there was a diagnostic delay of ~8 months. The majority of tumors of the base of the tongue are in advanced stages at diagnosis, while tumors of the mobile tongue that produce more functional alterations are usually diagnosed at an earlier stage (5,11).

ACCs have been histologically graded on the basis of their solid component: Grade 1, no solid component; grade 2, <30% solid component; and grade 3, ≥30% solid component (14–16). The solid subtype presents the worst prognosis, with a survival of 34% at 10 years, in contrast to 76% of the cribriform and 100% of the tubular subtypes, and is associated with recurrences, early distant metastases and a higher mortality rate (17). Other studies, however, did not found any correlation between microscopic appearance and prognosis (8). Franzén *et al* (14) reported that the prognosis is adversely effected by grade 3 histology (P=0.0001), DNA aneuploidy and an S-phase of ≥6% (P=0.0044) (14). Huang *et al* (18) observed a survival rate of 16.7 % after a 10-year treatment for cases where solid variable was observed and 47.4% for cribriform and tubular lesions. Norberg-Spaak *et al* (16) assessed mindbomb E3 ubiquitin protein ligase 1 (MIB1) positivity as an indicator of proliferation and found that the mean percentage of MIB1-positive cells was significantly correlated with the histological grade and, that the chance of recurrence was significantly lower for patients with low-MIB1 positivity.

The primary treatment objective in patients with ACC is local control, normal functionality and distant metastasis prevention and, for this purpose, early detection by the surgeon is required (3). Surgery is the cornerstone of treatment (5,11) and its extension depends on the size of the tumor. It may be conducted as a partial glossectomy, hemiglossectomy, total glossectomy with or without preservation of the larynx, or total glossectomy with or without reconstruction (11). A study by Soares *et al* (3) that was similar to the current study reported the case of a 64-year-old systemically healthy female patient who presented with a posterior tongue ACC. A hemiglossectomy and cervical lymph node emptying at the third level were carried out, with post-surgical radiotherapy as an additional treatment. Compared with other malignant neoplasms, ACC is more difficult to fully remove, with frequently identified positive surgical margins (3). Radiotherapy is indicated when surgical margins are compromised; the use of adjuvant radiotherapy remains controversial (11). Certain studies administered adjuvant radiotherapy following surgery in all patients regardless of the margin status, with an increase in the local control rate at 5 or 10 years (19,20). However, another study considered radiotherapy only for advanced T stages and as adjuvant in the presence of positive microscopic margins (21). Chen *et al* (22) found that the independent predictors of local recurrence were omission of postoperative radiotherapy (P=0.007), T4 disease (P=0.0001), perineural invasion (P=0.008) and major nerve involvement (P=0.02). Surgery and adjuvant radiotherapy [to doses in excess of 60 Gy, according to Chen *et al* (22)] offer the best chance of curing patients with resectable ACC of the head and neck. Certain patients with advanced, incompletely resectable disease can be treated with radiotherapy alone. Some minor salivary gland ACCs may be completely resected less often compared with those in the major salivary glands and have a lower probability of cure with radiotherapy alone (19). Comparison of the efficacy of radiotherapy alone vs. surgery and radiotherapy is effected by selection bias: Patients with early-stage lesions tend to undergo surgery, while those with advanced, unresectable cancers tend to be treated with radiotherapy alone (19). Miglianico *et al* (23) reported that the improvement in local-regional control could not result in a significant improvement in survival rates following surgery compared with combined modality therapy and that the lack of a survival advantage may be a result of the high rate of distant metastases.

Certain studies advocate adjuvant chemotherapy in addition to radiotherapy alone or combined with surgery due to the high risk of hematogenous dissemination, however, there is no convincing evidence that adjuvant chemotherapy is beneficial (24), as experience with adjuvant chemotherapy for patients with ACCs is limited (19). Concomitant chemo-radiotherapy is an alternative for patients with advanced disease or to preserve organs, but it is relatively inefficacious in treating ACC (11).

The probability of regional lymph node metastases in ACC of head and neck region is relatively low, ranging from 6 to 10% (5,23,25–27), and one of the reasons may be that the two commonest sites for ACC, parotid and palate/maxilla, have low propensity for nodal spread (27). Min *et al* (5), in a retrospective study involving 616 patients with ACC of the head and neck region, reported that lesions involving the base of the tongue, the mobile tongue and the mouth floor were the three primary sites with the highest incidences of cervical lymph nodes metastasis (19.2, 17.6 and 15.3%, respectively). Primary tumor site and peri-tumoral lymphovascular invasion were significantly associated with cervical lymph node metastasis. Levels Ib and II were the most frequently involved, with a reported incidence of 27 (43.5%) and 37 (59.6%) cases, respectively, while levels III and IV were affected only in 14 cases (22.5%). The overall survival rate at 5 years was significantly lower (P=0.037) in patients with lymph node metastasis (48%), compared with those without lymph node metastasis (77%). The authors proposed that selected neck dissection is not necessary in the majority of cases of primary cN0 patients. In addition, for floor-of-mouth and tongue lesions, dissection should be considered in patients with cN0 neck and larger ACC in these sites with lymphovascular invasion. Furthermore, for primary or recurrent cN+ patients, the combined approach of radical surgery, including wide resection of the original tumor and radical neck dissection, followed by postoperative irradiation, is strongly recommended (5). A previous study has suggested that it would be prudent to electively treat the first echelon nodes, particularly in patients with primary tumors in sites rich in lymphatic vessels, including the base of the tongue and nasopharynx (4). In accordance with these studies, the present study performed an elective emptying of cervical lymph nodes at levels I–III ipsilateral to the tumor. Balamucki *et al* (19) found that the rates of neck control at 5 and 10 years were 95 and 89% following observation and 98 and 98% subsequent to elective nodal irradiation, performed for tumors located in primary sites rich in capillary lymphatics. Gomez *et al* (28) reported that their patients who received radiotherapy to the neck experienced 100% neck control, while four of 50 node-negative patients experienced a neck failure at a median 12.6 years after radiotherapy.

ACC has a high propensity for perineural invasion (11,19), which indicates a worse prognosis, decreasing survival to 76% against 100% of those not presenting with it (17).

C-kit (CD-117), a tyrosine kinase receptor involved in the growth and development of normal tissues and in certain neoplasms, has been previously identified to be expressed in ACC (29). Luna-Ortiz *et al* (11) observed c-kit positivity in all their eight cases of ACC of the tongue, and it was also observed in the present case. Certain studies have considered the use of tyrosine kinase inhibitors, including imatinib mesylate, as an adjuvant and/or therapeutic tool in managing distant metastases. A study by Alcedo *et al* (30) identified favorable results in two cases of unresectable ACC treated with imatinib mesylate; one for recurrent disease and the other for a locally advanced tumor. However, it has been observed the progression of the disease during treatment with imatinib mesylate (31). Mutations in 11 c-kit and 9 c-kit exons condition an 83.5 and 47.8% response to tyrosine kinase inhibitors, respectively, in contrast to patients with non-detectable c-kit mutations, who do not present an objective response (32). The relevance of c-kit expression is due to its potential role in direct treatment of non-resectable or metastatic disease, although the use of imatinib mesylate remains controversial, which is also due to its high cost (11).

Other studies have revealed that a high proportion (85%) of ACCs stain positively for epidermal growth factor receptors (EGFR) and may respond to agents that act as EGFR inhibitors (33).

ACC presents a poor prognosis and it is therefore necessary to carry out long-term follow-up (Tables II and III). It has been reported to be a recurrent disease following treatment, ranging from 19 to 51% (19,25) with a median time to recurrence of 2.7 years (range, 0,3–20.2 years) (19). The incidence of ACC with distant metastasis, ranging from 31 to 50%, leads to a low long-term survival rate (5,11,23,25). The reported overall survival in ACC of the head and neck ranges from 51–56.5% and 32.5–34% at 5 and 10 years, respectively (11,34). In conclusion, the current study presents a noteworthy case of c-kit positive posterior tongue ACC treated with surgery and adjuvant radiotherapy. The cervical lymph nodes were also emptied ipsilateral to the tumor at levels I–III, as according to other studies the base of the tongue is a site rich in lymphatic vessels.

## Figures and Tables

**Table I:** Cases of ACC of the base of the tongue reported by Luna-Ortiz *et al* (9).

Age, years	Gender	T	N	M	Clinical stage	Surgery	Metastatic lymph nodes	Distant metastases	Follow-up
38	F	4	0	0	IVA	Left hemiglossectomy	No	No	Alive without disease after 207 months
48	F	-	-	-	Not classified due to being a recurrence	Total glossectomy, total laryngectomy, floor of mouth resection and reconstruction with pectoral flap	No	Bilateral pulmonary	Fatality with disease after 84 months
64	F	2	1	0	III	Hemiglossectomy, left base of tongue excision, supraomohyoid left neck dissection and reconstruction with pectoral flap	Yes, lymph node conglomerate	No	Fatality without disease after 84 months
40	F	4	0	X	IVA	Total glossectomy, floor of mouth resection and reconstruction with pectoral flap	No	Bilateral pulmonary	Fatality with disease after 60 months
67	M	3	0	0	III	Primary tumor resection, radical right neck dissection	2/39	No	Fatality without disease
63	F	3	0	0	III	Did not accept treatment	-	-	-

ACC, adenoid cystic carcinoma; T, tumor; N, node; M, metastasis; F, female; M (gender), male.

**Table II:** Local control rates: Review of the literature.

Authors, year (ref)	Local control rates, %					

	RT alone, years		Surgery alone, years		RT + surgery, years	
		
	5	10	5	10	5	10
Cowie *et al,* 1984 (11)	37				86	
Matsuba *et al,* 1984 (35)				25		83
Miglianico *et al,* 1987 (23)	66		44		78	
Balamucki *et al,* 2012 (19)	55	36			88	84

**Table III:** Survival: Review of the literature.

	RT alone, %		RT + surgery, %	
		
Authors, year (ref)	5 years	10 years	5 years	10 years
Overall survival				
Miglianico *et al,* 1987 (23)	79		72	
Balamucki *et al,* 2012 (19)	56	37	75	57
Disease-free survival				
Miglianico *et al,* 1987 (23)	47		54	
Balamucki *et al,* 2012 (19)	65	46	79	71
Distant metastasis-free survival				
Balamucki *et al,* 2012 (19)	80	76	72	62

## References

[1] Billroth T (1859). Observations on tumors of the salivary glands. Virchows Arch Pathol Anat.

[2] Foote FW, Frazell EL (1953). Tumors of the major salivary glands. Cancer.

[3] Soares EC, Carreiro Filho FP, Costa FW, Vieira AC, Alves AP (2008). Adenoid cystic carcinoma of the tongue: case report and literature review. Med Oral Patol Oral Chir Bucal.

[4] Kim KH, Sung MW, Chung PS (1994). Adenoid cystic carcinoma of the head and neck. Arch Otolaryngol Head Neck Surg.

[5] Min R, Siyi L, Wenjun Y, Ow A, Lizheng W, Minjun D, Chenping Z (2012). Salivary gland adenoid cystic carcinoma with cervical limph node metastasis: a preliminary study of 62 cases. Int J Oral Maxillofac Surg.

[6] Spiro RH (1986). Salivary neoplasms: overview of a 35-year experience with 2,807 patients. Head Neck Surg.

[7] Khafif A, Anavi Y, Haviv J, Fienmesser R, Calderon S, Marshak G (2005). Adenoid cystic carcinoma of the salivary glands: a 20-year review with long-term follow-up. Ear Nose Throat J.

[8] Spiro RH, Huvos AG, Strong EW (1974). Adenoid cystic carcinoma of salivary origin. A clinicopathologic study of 242 cases. Am J Surg.

[9] Triantafillidou K, Dimitrakopoulos J, Iordanidis F, Koufogiannis D (2006). Management of adenoid cystic carcinoma of minor salivary glands. J Oral Maxillofac Surg.

[10] Cowie VJ, Pointon RC (1984). Adenoid cystic carcinoma of the salivary glands. Clin Radiol.

[11] Luna-Ortiz K, Carmona-Luna T, Cano-Valdez AM (2009). Adenoid cystic carcinoma of the tongue - clinicopathological study and survival analysis. Head Neck Oncol.

[12] van der Wal JE, Becking AG, Snow GB, van der Waal I (2002). Distant metastases of adenoid cystic carcinoma of the salivary glands and the value of diagnostic examinations during follow-up. Head Neck.

[13] Namazie A, Alavi S, Abemayor E, Calcaterra TC, Blackwell KE (2001). Adenoid cystic carcinoma of the base of the tongue. Ann Otol Rhinol Laryngol.

[14] Franzén G, Nordgård S, Boysen M, Larsen PL, Halvorsen TB, Clausen OP (1995). DNA content in adenoid cystic carcinomas. Head Neck.

[15] Luna MA, el-Naggar A, Batsakis JG, Weber RS, Garnsey LA, Goepfert H (1990). Flow cytometric DNA content of adenoid cystic carcinoma of submandibular gland. Correlation of histologic features and prognosis. Arch Otolaryngol Head Neck Surg.

[16] Norberg-Spaak L, Dardick I, Ledin T (2000). Adenoid cystic carcinoma: use of cell proliferation, BCL-2 expression, histologic grade, and clinical stage as predictors of clinical outcome. Head Neck.

[17] Sequeiros Santiago G, Rodrigo Tapia JP, Llorente Pendás JL, Suárez Nieto C (2005). Prognostic factors in adenoid cystic carcinoma of salivary glands. Acta Otorrinolaringol Esp.

[18] Huang M, Ma D, Sun K, Yu G, Guo C, Gao F (1997). Factors influencing survival rate in adenoid cystic carcinoma of the salivary glands. Int J Oral Maxillofac Surg.

[19] Balamucki CJ, Amdur RJ, Werning JW (2012). Adenoid cystic carcinoma of the head and neck. Am J Otolaryngol.

[20] Mendenhall WM, Morris CG, Amdur RJ (2004). Radiotherapy alone or combined with surgery for adenoid cystic carcinoma of the head and neck. Head Neck.

[21] Silverman DA, Carlson TP, Khuntia D, Bergstrom RT, Saxton J, Esclamado RM (2004). Role for postoperative radiation therapy in adenoid cystic carcinoma of the head and neck. Laryngoscope.

[22] Chen AM, Bucci MK, Weinberg V (2006). Adenoid cystic carcinoma of the head and neck treated by surgery with or without postoperative radiation therapy: prognostic features of recurrence. Int J Radiat Oncol Biol Phys.

[23] Miglianico L, Eschwege F, Marandas P, Wibault P (1987). Cervico-facial adenoid cystic carcinoma: study of 102 cases. Influence of radiation therapy. Int J Radiat Oncol Biol Phys.

[24] Diaz EM, Kies MS (2001). Chemotherapy for skull base cancers. Otolaryngol Clin North Am.

[25] Garden AS, Weber RS, Morrison WH, Ang KK, Peters LJ (1995). The influence of positive margins and nerve invasion in adenoid cystic carcinoma of the head and neck treated with surgery and radiation. Int J Radiat Oncol Biol Phys.

[26] Khan AJ, DiGiovanna MP, Ross DA, Sasaki CT, Carter D, Son YH, Haffty BG (2001). Adenoid cystic carcinoma: a retrospective clinical review. Int J Cancer.

[27] Oplatek A, Ozer E, Agrawal A, Bapna S, Schuller DE (2010). Patterns of recurrence and survival of head and neck adenoid cystic carcinoma after definitive resection. Laryngoscope.

[28] Gomez DR, Hoppe BS, Wolden SL (2008). Outcomes and prognostic variables in adenoid cystic carcinoma of the head and neck: a recent experience. Int J Radiat Oncol Biol Phys.

[29] Andreadis D, Epivatianos A, Poulopoulos A, Nomikos A, Papazoglou G, Antoniades D, Barbatis C (2006). Detection of C-KIT (CD117) molecule in benign and malignant salivary gland tumours. Oral Oncol.

[30] Alcedo JC, Fábrega JM, Arosemena JR, Urrutia A (2004). Imatinib mesylate as treatment for adenoid cystic carcinoma of the salivary glands: report of two successfully treated cases. Head Neck.

[31] Lin CH, Yen RF, Jeng YM, Tzen CY, Hsu C, Hong RL (2005). Unexpected rapid progression of metastatic adenoid cystic carcinoma during treatment with imatinib mesylate. Head Neck.

[32] Hotte SJ, Winquist EW, Lamont E (2005). Imatinib mesylate in patients with adenoid cystic cancer of the salivary gland expressing c-kit: A Princess Margaret Hospital phase II consortium study. J Clin Oncol.

[33] Vered M, Braunstein E, Buchner A (2002). Immunohistochemical study in epidermal growth factor receptor in adenoid cystic carcinoma of salivary gland origin. Head Neck.

[34] da Cruz Perez DE, de Abreu Alves F, Nobuko Nishimoto I, de Almeida OP, Kowalski LP (2006). Prognostic factors in head and neck adenoid cystic carcinoma. Oral Oncol.

[35] Matsuba HM, Thawley SE, Simpson JR, Levine LA, Mauney M (1984). Adenoid cystic carcinoma of major and minor salivary gland origin. Laryngoscope.

